# Editorial: Pharmacogenetics Research and Clinical Applications: An International Landscape of the Accomplishments, Challenges, and Opportunities

**DOI:** 10.3389/fphar.2020.01217

**Published:** 2020-08-14

**Authors:** Nathalie K. Zgheib, George P. Patrinos

**Affiliations:** ^1^ Department of Pharmacology and Toxicology, American University of Beirut, Beirut, Lebanon; ^2^ Department of Pharmacy, University of Patras School of Health Sciences, Patras, Greece; ^3^ Department of Pathology, College of Medicine and Health Sciences, United Arab Emirates University, Al-Ain, United Arab Emirates; ^4^ Zayed Center of Health Sciences, United Arab Emirates University, Al-Ain, United Arab Emirates

**Keywords:** pharmacogenetics (PGN), pharmacogenomics (PGx), research, clinical, challenges, international

Pharmacogenomics (PGx) is a major pillar of personalized medicine. With the effort to tailor therapeutic interventions to patients depending on their genetic profile came a higher demand for further PGx research. As naturally expected, discrepancies have been noted, with some institutions leading the way in this field. The majority of the documented efforts in research and clinical applications are concentrated mostly in the US and Europe, while relatively little information is available from other parts of the world (Abou Diwan et al., 2019; Zgheib et al., 2020). Therefore, the goal of this Research Topic is to shed more light onto worldwide accomplishments in PGx research and clinical applications with a focus on current challenges, lessons learned, and opportunities for further advances in the field towards better clinical uptake of PGx.

This issue includes 13 contributions to the field emanating from 11 countries scattered thorough the globe and spanning five continents. The data presented are quite representative of the status of PGx research worldwide, and relate to the following topics: validation of already established or extensively studied PGx markers in new populations, frequency distribution of actionable pharmacogenes in multiethnic groups, attempts at the discovery of novel PGx markers that are peculiar to certain populations, evaluations of the implementation of PGx guided practice, perspectives of the challenges of PGx applications in the clinic, and economic evaluation of various reimbursement models for PGx testing.

Perhaps one of the most commonly studied drug-gene pairs is that of oral anticoagulants such as acenocoumarol and warfarin with *CYP2C9*, *CYP4F2*, and *VKORC1* candidate polymorphisms (Johnson et al., 2017). Nevertheless, the published guidelines may not necessarily apply to all populations, hence the need for validation studies in different contexts. As such, three manuscripts address the issue from three different angles. Roco et al. provide a pharmacogenetically guided algorithm that explains almost 50% of the variability of acenocoumarol dosing in Chilean patients. Roche-Lima et al. compare and contrast seven machine learning algorithms for the prediction of warfarin dosing from PGx data and conclude that Random Forest Regression (RFR) outperforms all other models. Finally, Zhang et al. show that gene-based warfarin dosing provides clinical benefits in Chinese patients when compared with clinically fixed dosing.

The PGx of thiopurines is another topic that has been extensively studied, and updated guidelines for preemptive genotyping for *Thiopurine-S-methyltransferase (TPMT)* and *NUDT15* genetic polymorphisms have been published recently (Relling et al., 2019). Nevertheless, the minor allele frequencies of polymorphisms in these genes vary among populations and may be quite rare in, for example, Middle Eastern populations. Accordingly, Moradveisi et al. reveal that two variants in *Inosine triphosphatase (ITPA)* might also be relevant in predicting 6-mercaptopurine toxicity in Arab and Kurdish children treated for Acute Lymphoblastic Leukemia.

The results described above highlight the importance of identifying and characterizing both novel and previously known PGx variants in various populations. As such, Galaviz-Hernandez et al. report on the frequency of CYP3A5*3 allele in eight different ethnic groups from Northwest Mexico and its association with hypertension. Another study by Gonzalez-Covarrubias et al. characterizes variations in actionable PGx markers in 1,284 Mestizos and 94 natives from Mexico. Of note in the latter study is the multi-institutional collaborative aspect of the endeavor that allowed the investigators to access a large number of samples with deep sequencing data (Gonzales-Covarrubias et al.). A relatively small sample size may be an issue in gene discovery studies, especially if dealing with relatively less common phenotypes or in countries of lower income. For example, an evaluation of 15 variants in *Reelin (RELN)* shows that two novel loci may be associated with response to antipsychotics in 260 Chinese Hans, yet, the statistical significance was lost after multiple corrections (Xu et al.). Similarly, another study, the first of its kind to explore innate immune genetic polymorphisms in 154 patients treated with tacrolimus for kidney transplant in Australia, also shows loss of statistical significance after Bonferroni adjustment (Hu et al.). On the other hand, another evaluation of a much smaller number (N = 76) of kidney transplant patients from Egypt indicates that the common CYP3A5*3 polymorphism is associated with tacrolimus daily requirements in these patients (Mendrinou et al.).

While some countries are still investigating the effects or frequencies of PGx markers in their populations, others with higher income such as The Netherlands and Singapore are at stages of evaluating the implementation of clinical practice guidelines in their settings (Martens et al.; Rigter et al.; Sung et al.). More specifically in The Netherlands, a multi-stakeholder perspective of the implementation of PGx in primary care suggests that, despite the ability to formulate actions to truly integrate PGx, there is no consensus on the prioritization of these actions (Rigter et al.). Furthermore, Martens et al. disclose that the frequency of *DPD* testing before initiation of fluoropyrimidine treatment for patients with colon cancer significantly increased only after the update of a National guideline and local consensus meetings. Along the same lines in Singapore, the incidence of severe cutaneous reactions in association with carbamazepine was significantly decreased after the issuing of National recommendations for HLA-B*15:02 genotyping (Sung et al.). These results undoubtedly affirm the essential role of practice guidelines for the clinical applications of PGx testing; nevertheless, the cost of these tests may be problematic especially if not or only partially reimbursed. As such, Simeonidis et al., who undertook a very elegant systematic analysis and economic evaluation to assess the feasibility of compensation for PGx testing, conclude that such data are lacking from the literature, hence the need for more cost-utility analyses within various healthcare systems.

In summary, the compilation of manuscripts in this Research Topic gives a taste of the ongoing PGx research and clinical applications worldwide, especially in developing countries. Further studies are needed to cover more diverse populations and ethnicities and to unravel the challenges and solutions to make personalized medicine a global reality (Zgheib et al., 2020).

## Author Contributions

All authors listed have made substantial, direct, and intellectual contribution to the work and approved it for publication.

## Conflict of Interest

The authors declare that the research was conducted in the absence of any commercial or financial relationships that could be construed as a potential conflict of interest.
